# Neighborhood Emission Mapping Operation (NEMO): A 1-km anthropogenic emission dataset in the United States

**DOI:** 10.1038/s41597-022-01790-9

**Published:** 2022-11-09

**Authors:** Siqi Ma, Daniel Q. Tong

**Affiliations:** Department of Atmospheric, Oceanic and Earth Sciences, George Mason University, Fairfax, VA 22030 USA

**Keywords:** Atmospheric chemistry, Sustainability

## Abstract

We present an unprecedented effort to map anthropogenic emissions of air pollutants at 1 km spatial resolution in the contiguous United States (CONUS). This new dataset, Neighborhood Emission Mapping Operation (NEMO), is produced at hourly intervals based on the United States Environmental Protection Agency (US EPA) National Emission Inventories 2017. Fine-scale spatial allocation was achieved through distributing the emission sources using 108 spatial surrogates, factors representing the portion of a source in each 1 km grid. Gaseous and particulate pollutants are speciated into model species for the Carbon Bond 6 chemical mechanism. All sources are grouped in 9 sectors and stored in NetCDF format for air quality models, and in shapefile format for GIS users and air quality managers. This dataset shows good consistency with the USEPA benchmark dataset, with a monthly difference in emissions less than 0.03% for any sector. NEMO provides the first 1 km mapping of air pollution over the CONUS, enabling new applications such as fine-scale air quality modeling, air pollution exposure assessment, and environmental justice studies.

## Abstract (summary)

**Table:** 

Measurement(s)	anthropogenic emission in United States
Technology Type(s)	Census survey and computer models

## Background & Summary

Emission, or the release of gases and particles from the Earth’s surface into the atmosphere, is the starting point of many Earth system processes responsible for some of the greatest environmental challenges today, such as air pollution, acid deposition, and climate change^1–3^. The World Health Organization (WHO) estimates that exposure to ambient air pollution has been associated with 7 million premature deaths per annum, making it the single largest environmental risk today^4^. In the United States, over one third of the population lives in areas not attaining the health-based National Ambient Air Quality Standards (NAAQS) for ozone (O_3_) and/or fine particulate matter (PM_2.5_)^5^. Air quality and public health managers have an important task to protect public health by alerting the population when forecasts predict the exceedance of the NAAQS, which critically depends on the accurate prediction of the timing, location, and severity of unhealthy air quality episodes^6,7^.

Air quality models used for forecasting and policy studies rely on detailed mapping of emission sources to predict spatio-temporal variations of air pollution. Air pollutants can be directly emitted (primary) or formed in the atmosphere through chemical/physical processes (secondary)^8^. While PM_2.5_ can have both primary and secondary origins, O_3_ is mostly formed through photochemical reactions in the troposphere^9,10^. Consequently, the collocation of emitted pollutants and their precursors, that affects the chemical transformations, localized dispersion and deposition, is a major factor controlling the variability of concerned atmospheric constituents^11–13^. Better spatially resolved emission data allows further improvement of atmospheric composition prediction for air quality early warning and management^14–16^. Chemical transport models can provide the knowledge of vertical atmospheric constituents as priori for the retrievals of satellite products^17–20^. As technology advances, the instruments are able to observe at higher spatial resolution, requiring a priori information at a finer resolution as well. Similarly, fine resolution emission and concentration data can provide new insight into population exposure to air pollution^21–23^.

Numerous approaches have been utilized to map greenhouse gases and air pollutants at high spatial resolution. A global CO_2_ emission dataset with 1 km resolution was developed by using the satellite observed nighttime lights^24^, and a global ammonia emission inventory with 0.1° resolutions was created with updated emission factors and data products^25^. Within the US, the DAtabase of Road Transportation Emissions (DARTE) provides annual emissions of on-road CO_2_ over the contiguous United State (CONUS) at 1 km resolution based on the roadway-level and emission factors^26^. The Vulcan v3.0 CO_2_ emissions data was generated at 1 km and hourly resolution which includes various anthropogenic source sectors^27^. Additionally, a sub-neighborhood (~100 m) surface NO_2_ dataset over the CONUS was presented using land-use regression (LUR) models along with observational and modeling data^28,29^. These datasets usually focus on a single emission species, and are often inadequate for some applications, such as air quality modeling. A high-resolution emission dataset that contains all co-emitted major air pollutants is desirable to support various applications in air quality modeling, public health, and environmental management.

In this study, we present a new high‐resolution anthropogenic emission dataset, called the Neighborhood Emission Mapping Operation (NEMO), that maps all major sources in the CONUS. It includes the emissions from nine sectors, accounting for 854 individual source types, based on the 2017 National Emissions Inventory (NEI). The emission data are mapped at 1 km spatial gridding and at hourly intervals. These air pollutants are further split into chemical species consistent with the Carbon Bond 6 (CB6) chemical mechanism, so that the data can be used to drive air quality models such as the Community Multiscale Air Quality (CMAQ) model^30^ and the Weather Research and Forecast with chemistry (WRF-chem) model. The data are available in the NetCDF format, and annual data are also provided in the shapefile format for VOCs, NO_x_, CO, SO_2_, NH_3,_ and PM_2.5_. In addition, a web-based data portal has been set up to provide online emission data services for interested users. This, to our knowledge, is the first effort to map all major air pollutants at 1 km resolution for the entire CONUS. The dataset, along with the data access, is expected to enable new applications such as fine-scale air quality modeling, air pollution exposure assessment, and environmental justice studies.

## Methods

The anthropogenic emissions in this dataset are generated based on the 2017 National Emissions Inventory (NEI2017) from the US Environmental Protection Agency (US EPA). Since the NEI only provides aggregate emissions for each county, four steps were taken to generate the high-resolution emission dataset, including 1) spatial allocation; 2) chemical speciation; 3) temporal allocation, and 4) merging. These four steps are implemented by the Sparse Matrix Operator Kernel Emissions (SMOKE) model^31^, and the configuration files (usually called profiles) used for the allocation and speciation in SMOKE can be generated with various tools or provided by the ancillary datasets from US EPA. The information of the emission inventory and other data and tools is described as follows.

### Base emission inventories

The NEI2017 (version 2017gb) compiled by US EPA is used to develop the high-resolution emission dataset. There are hundreds of individual emission sources in NEIs, which are grouped into nine emission sectors, including six nonpoint sectors, two mobile sectors, and the point sector (Table 1). Each emission source is identified by a unique source classification code (SCC). Except the point source, all sources are provided at county level. For each county, the NEI lists the annual amounts of emitted air pollutants, including fine and coarse particulate matter (PM_2.5_ and PM_10_), nitrogen oxides (NO_x_), carbon monoxide (CO), sulfur dioxide (SO_2_), ammonia (NH_3_), and volatile organic compounds (VOCs). Point sources are represented as the individual facilities (energy, industrial, and manufacturing facilities), usually at specific latitude/longitude coordinates, rather than as county or tribal aggregates. In NEI2017, all point sectors are treated as elevated sources, so in this dataset we only consider the airports sector, which has surface level of emissions and can be processed into two-dimension gridded files. The Motor Vehicle Emissions Simulator (MOVES) version 2014b generates county-level emission factors from on-road mobile sources, which include monthly county-level emissions from motorized vehicles that are normally operated on public roadways. In addition, emissions from nonroad sources, such as nonroad engines and equipment, construction equipment, and agricultural engines, are also calculated by the nonroad component of the EPA’s MOVES model (MOVES-Nonroad). For the estimated emission records, quality assurance (QA) has been implemented and reviewed by EPA and state, local, and tribal agencies. Detailed information about the emission inventory is provided in the NEI2017 Technical Support Document (TSD)^32^ and all the inventory files, as well as the emission processing platform, can be downloaded from EPA FTP^33^.

**Table 1:** Overview of NEI2017 inventory used for the emission processing.

Sector	Type	Sector Description	Spatial/Temporal resolution	Related surrogate code
afdust	nonpoint	Anthropogenic fugitive dust emissions	County/Annual	240, 304, 306, 308, 310, 340
ag	nonpoint	Agricultural ammonia sources	County/Annual	100, 310
nonpt	nonpoint	Nonpoint sources not in other sectors	County/Annual	100, 150, 170, 180, 190, 239, 240, 244, 271, 300, 306, 307, 308, 310, 319, 320, 505, 535, 650, 711, 801
np_oilgas	nonpoint	Nonpoint oil and gas-production-related sources	County/Annual	670, 671, 672, 674, 678, 679, 681, 683, 685, 687, 691, 692, 693, 694, 695, 696, 697, 698, 699
rail	nonpoint	Locomotive sources on railroads	County/Annual	100, 261, 271
rwc	nonpoint	Residential wood combustion sources	County/Annual	100, 300
non-road	mobile	On-land mobile sources not on roads or railroads	County/Monthly	100,261, 304, 305, 306, 307, 308, 309, 310, 320, 321, 350, 850, 860
on-road	mobile	On-land mobile sources that drive on roads	County/Monthly	100, 242, 244
airports	point	Airport emissions	Lonlat/Annual	NEI Latitude/Longitude

### Chemical speciation

Some of the pollutants (namely NO_x_, VOCs, PM_2.5_ and PM_10_) in the emission inventory cannot be directly used by chemical transport models, unless distributed into model species of a specific chemical mechanism. The model species can be individual chemical compounds (explicit species) or groups of species (lumped species). In the NEI2017, we use the Carbon Bond 6 (CB6) chemical mechanism^34^ to split gaseous pollutants (NOx and VOCs), and the Aerosol 7 (AERO7) aerosol mechanism^35^ to split particulate pollutants (PM_2.5_ and PM_10_) into required model species.

Chemical speciation of the pollutants is achieved using detailed chemical profiles that allocate an aggregate pollutant to required model species. For VOCs, the speciation profiles generally have two types, “CRITERIA” and “INTEGRATE”. “CRITERIA” means all model species are speciated from the total VOC emissions in NEI. This VOC speciation approach is applied to point sources and several area sources that are not included in the Hazardous Air Pollution (HAP) inventory. The other VOC speciation approach, called Integration or “INTEGRATE”, is used for onroad, offroad and some area source sectors. This approach aims to integrate two NEIs, NEI2017 and HAP NEI, for select VOC HAPs. For these HAPs, the HAP NEI is generally considered a better data source than speciated VOC in NEI2017. Five VOC HAPs, including naphthalene (NAPH), benzene (BENZ), acetaldehyde (ALD2), formaldehyde (FORM) and methanol (MEOH) collectively called NBAFM, are explicitly represented in the CB6 chemical mechanism. The “INTEGRATE” profiles are used to subtract NBAFM from the total VOC during the speciation processes to avoid double counting emissions. For instance, in the airports sector, the NEI2017 provides the total VOC emission named as “VOC” and no integration is needed for the chemical speciation. All the model species are speciated from the “VOC” in the NEI2017. In contrast, the onroad and offroad emission inventory provides specific emissions for HAP species (i.e., NBAFM) and the VOC emissions that exclude those species. Therefore, these HAP will be removed from the criteria VOC mass, and the profiles are generated by removing the specified HAP species from the “CRITERIA” profiles, and then renormalizing. Detailed information of the use of HAP along with NEI VOC, called “HAP-CAP integration”, and the integration status for each emission sector can be found in the Table 3-4 of the TSD for the 2016 NEI Collaborative^36^.

The speciation profiles for most emission sectors can be created by the Speciation Tools^37^ on the basis of SPECIATE database^38^ which is developed and maintained by the Office of Research and Development (ORD) of US EPA. The only exception is that the speciation profiles of the mobile sources (on-road and non-road sectors, other than for California) are generated by the Motor Vehicle Emissions Simulator (MOVES)^39^. Similar to the VOC, the speciation information of PM is also supported by the SPECIATE and can be generated using the Speciation Tools and MOVES. For NO_x_, the speciation is based on a NO_2_ weight factors, speciating total NO_x_ into NO, NO_2_, and/or HONO. The speciate profiles for different emission sources and locations are differentiated by the SCC and county/state, managed through a cross-reference file that links SCC for each county/state to a specific speciation profile. In NEI2017, the speciation profiles for the CB6 mechanism are already prepared by EPA, which are created based on the SPECIATE5.0 database^33^.

### Temporal allocation

NEI provides annual totals but models require the information of finer temporal variations (monthly, weekly, daily and hourly). Distributing aggregated emissions to a finer (hourly) temporal resolution to meet the model requirement is realized by the temporal allocation process. For the source sectors with annual emission records (Table 1), three temporal allocation profiles (annual-to-month, month-to-day, and diurnal) are applied. For the sectors with monthly emission records, the annual-to-month allocation will not be used. The temporal allocations are also based on the profile files which are obtained in several ways. The temporal profiles of most sectors are created based on the operational data from different agencies/industries, such as the Federal Aviation Administration (FAA) operations and performance data for airports sector and Association American Railroads (AAR) Rail Traffic data for rail sector. For some sectors, the temporal variations of the emissions are also controlled by meteorological conditions. Therefore, the meteorology-based temporal profiles are developed using a tool called “gentpro” using the weather data. These weather-adjusted profiles are applied to three sectors: anthropogenic fugitive dust, residential wood combustion, and agriculture. The temporal allocation of on-road sources is based on a combination of traditional temporal profiles and the influence of meteorology. The on-road inventory used in this study is in the Flat File 2010 (FF10) format processed from the MOVES outputs; therefore, the temporal profiles for this format are derived from MOVES and supported in the platform^33^. The temporal profiles for each source and county/state are assigned using a cross-reference file that links Federal Information Processing System (FIPS) code/SCC/pollutant to different monthly/weekly/diurnal temporal profiles.

### Spatial distribution

A major challenge to develop a neighborhood level emission dataset is how to spatially distribute the county-level emission aggregate from NEI into locations at finer scale. In this study, county-level emissions from nonpoint and mobile sources are spread among the grid cells intersecting the county by using spatial distribution profiles (namely spatial surrogates). A spatial surrogate ratio is a value greater than zero and less than or equal to unity that specifies the fraction of the emissions in an area (usually a county) that should be allocated to a particular model grid cell (a 1 km^2^ square in this case). As the area of a given county may fall into several grid cells, spatial surrogates need to be used to indicate the fraction of the county’s emissions assigned to each grid cell. These surrogates are created based on geographic information systems (GIS) shapefiles which include the geographic information, such as population/housing, roadways, and land cover (Supplementary Table 1) which act as weight factors when calculating different types of surrogate ratios. A spatial surrogate ratio file includes the grid description, surrogate code, FIPS, column/row number of the model grid, and spatial surrogate ratio (spatial factor).

In this study, the spatial surrogates for the 1 km × 1 km grids were generated using a surrogate generating tool Spatial Allocator (SA) coupled with the PostgreSQL database management system. The SA, developed by the University of North Carolina Community Modeling and Analysis System (CMAS), is a suite of tools to create input files for weather and air quality models. More specifically, the surrogate tools of SA were used to create a large set of spatial surrogates, and to merge and gap-fill these surrogates when necessary. The source code and scripts, as well as detail documentation of the SA tools can be downloaded from the CMAS center^40^. The procedures can be summarized in five steps: (1) Install the Spatial Allocator^41^ along with PostgreSQL software, and collect shapefile data from the EPA^42^ or commercial vendors; (2) Activate PostgreSQL server, create a database and load the shapefile data into database; (3) Generate a table representing the modeling grid in the database; (4) Generate surrogate files using SA tools; (5) Gap-filling, normalization, and quality assurance. For the contiguous United States (CONUS), a total of 108 spatial surrogates were prepared, including 12 U.S. census-based surrogates, 24 transportation surrogates (roadways, railways, bus terminals and idling), 17 landcover surrogates, 20 surrogates for building footprints, 23 surrogates that describe oil and gas well production, 6 surrogates for shipping and ports, and 6 for other industrial and commercial activities like refineries and tank farms, airports, golf courses, mines, and timber. The surrogate information and relevant shapefile data used for our dataset are provided in Supplementary Table 1.

### Generating 1 km emission dataset

With the base emission inventories, chemical speciation, temporal profiles and spatial surrogate ratios, we generate the 1 km emission dataset using the SMOKE model version 4.7 for all nine anthropogenic emission sectors. This process takes four steps. First, the chemical profiles are used to speciate NO_x_, VOCs, PM_2.5_ and PM_10_ into required chemical species for each source/location. Next, all emission records are distributed to 1-hour intervals from the 2017 annual or monthly total emissions using SCC‐specific temporal profiles. Third, the spatial surrogate ratios are used to distribute county-level emissions into 1 km × 1 km grids. Finally, all gridding, speciation, and temporal matrices are combined to create model-ready emission data at 1 km horizontal resolution and hourly intervals in the netCDF format.

**Fig. 1:**
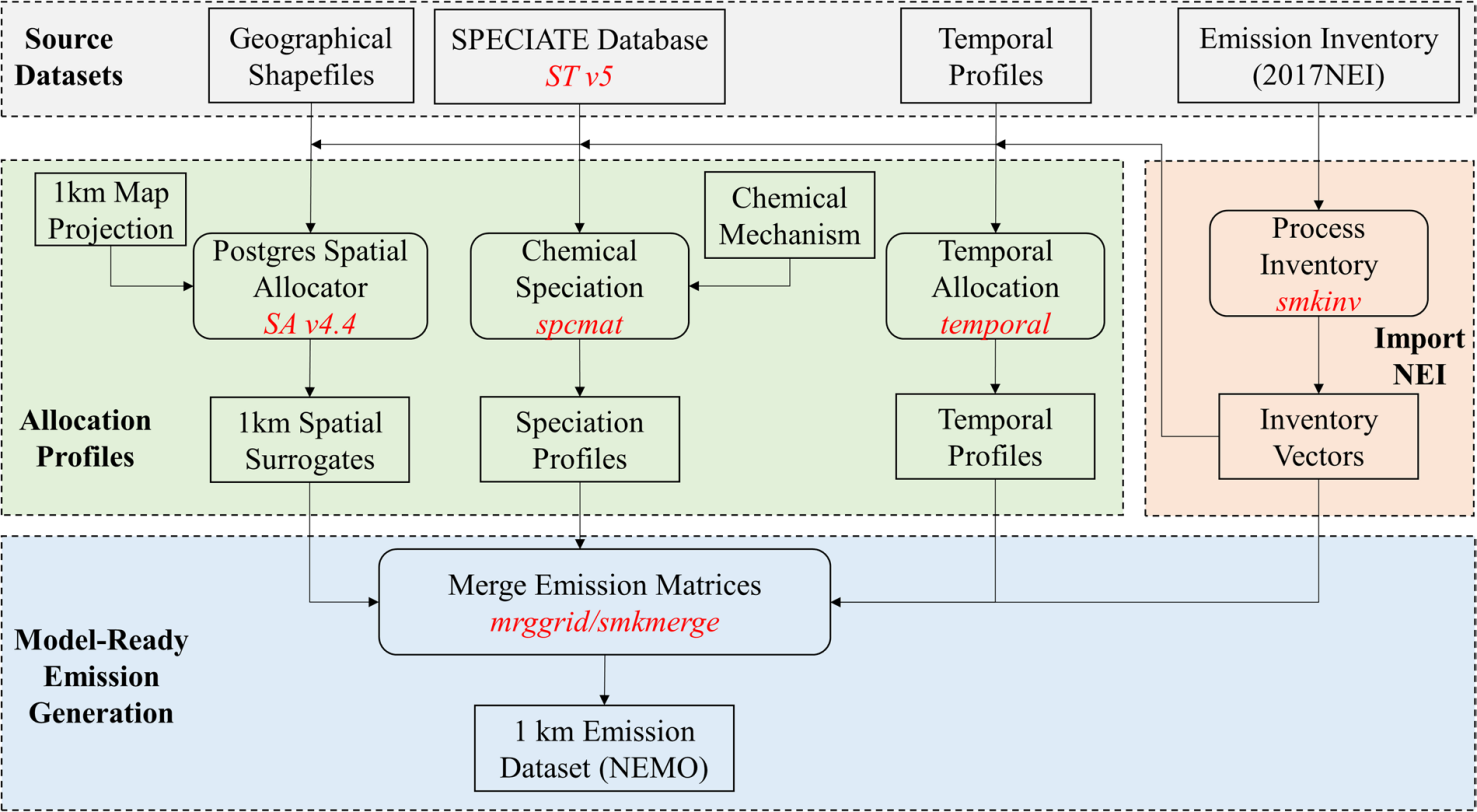
Procedures to generate the 1 km NEMO emission dataset. The rectangles represent input/output files, and the rounded-corner rectangles indicate the tools/programs/models used for the processes. SA v4.4 is the Spatial Allocator version 4.4, a tool used to create spatial surrogates. ST v5 is the Speciation Tools version 5 used to create chemical speciation profiles. The other programs, including spcmat, temporal, smkinv, mrggrid and smkmerge, are tools provided by the Sparse Matrix Operator Kennel Emission (SMOKE) package.

For each of the emission sectors, the above processes are repeated, so that the combined datasets are generated for each sector. The gridded emission will be stored by sectors and can be merged using a SMOKE tool (mrggrid) as needed, depending on the needs of the model simulation. The flow chart in Fig. 1 depicts the steps for generating the emission data and Table 1 shows the emission sectors that this dataset includes. For the all-sector merged emission data, we also convert the data into the Shapefile format, so that users may be able to visualize the data along with other maps (such as highways and street maps).

## Data Records

**Fig. 2:**
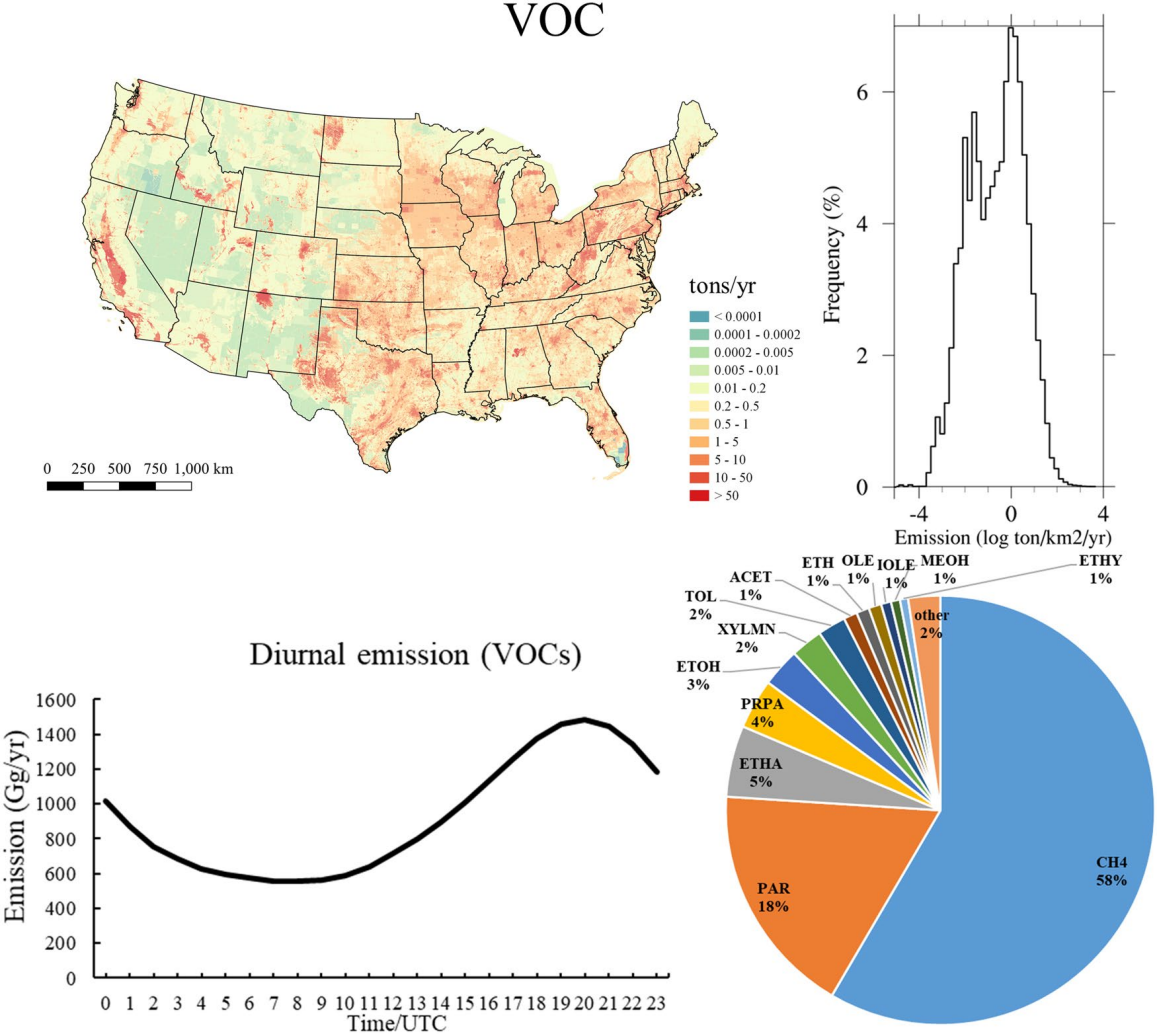
Annual emission of volatile organic compounds (VOC), their emission distribution frequency diagrams, diurnal variations, and portions of speciated model species of total VOC. The data are generated based on NEI2017.

**Fig. 3:**
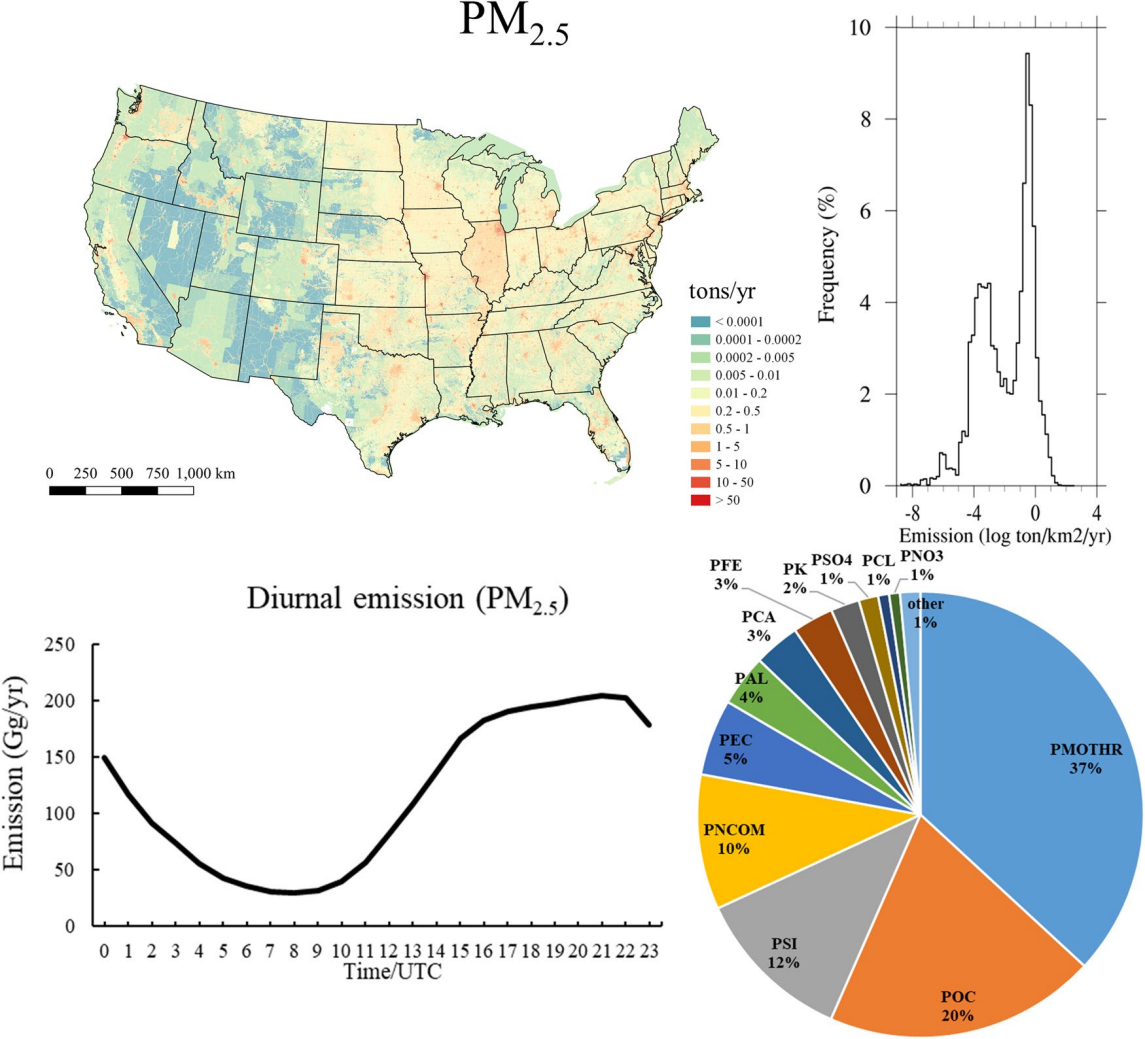
Annual emission of fine particulate matter (PM_2.5_), their emission distribution frequency diagrams, diurnal variations, and portions of speciated model species. The data are generated based on NEI2017.

Table 2 summarizes the information of the generated 1 km emission dataset. This emission dataset is stored in two formats: NetCDF for modeling and analysis, and Shapefile for use with GIS software. Both formats have the same emission sectors with 1 km^2^ resolution. The NetCDF format contains hourly, monthly, and annual data while the Shapefiles only include annual emissions. Additionally, the NetCDF provides the model species for CB6 mechanism in the hourly and monthly data files while the shapefiles include integrated species like VOCs, NO_x_, PM_2.5,_ and three inorganic gases, SO_2_, CO, and NH_3_. Figures 2 and 3 shows the example of the annual emission distributions of VOC and PM_2.5_, along with their frequency diagrams, as well as the diurnal variations and the proportions of each speciated model species from VOC and PM_2.5_. The datafiles of monthly and annual emissions that are available on figshare^43^, while the hourly emission data are stored on our data server at George Mason University^44^ because of the large file sizes.

**Table 2:** Information of NEMO emission dataset.

Feature	NEMO Dataset 1	NEMO Dataset 2
Format	NetCDF	shapefile
Base year	2017	
Sectors	Anthropogenic fugitive dust (afdust), agriculture (ag), non-point (nonpt), oil and gas operations (np_oilgas), onroad, nonroad, rail, residential wood combustion (rwc), and airports	
Temporal resolution	hourly, monthly, annual	annual
Spatial resolution	1 km × 1 km	
Grid projection	Lambert Conformal Conic projection	
Variables	Model species for Carbon bond 6 (hourly and monthly), VOCs, NO_x_, SO_2_, CO, NH_3_, PM_2.5_ (annual)	VOCs, NO_x_, SO_2_, CO, NH_3_, PM_2.5_
Supported models	CMAQ, WRF-chem

## Technical Validation

### Comparison with the EPA benchmark dataset

**Fig. 4:**
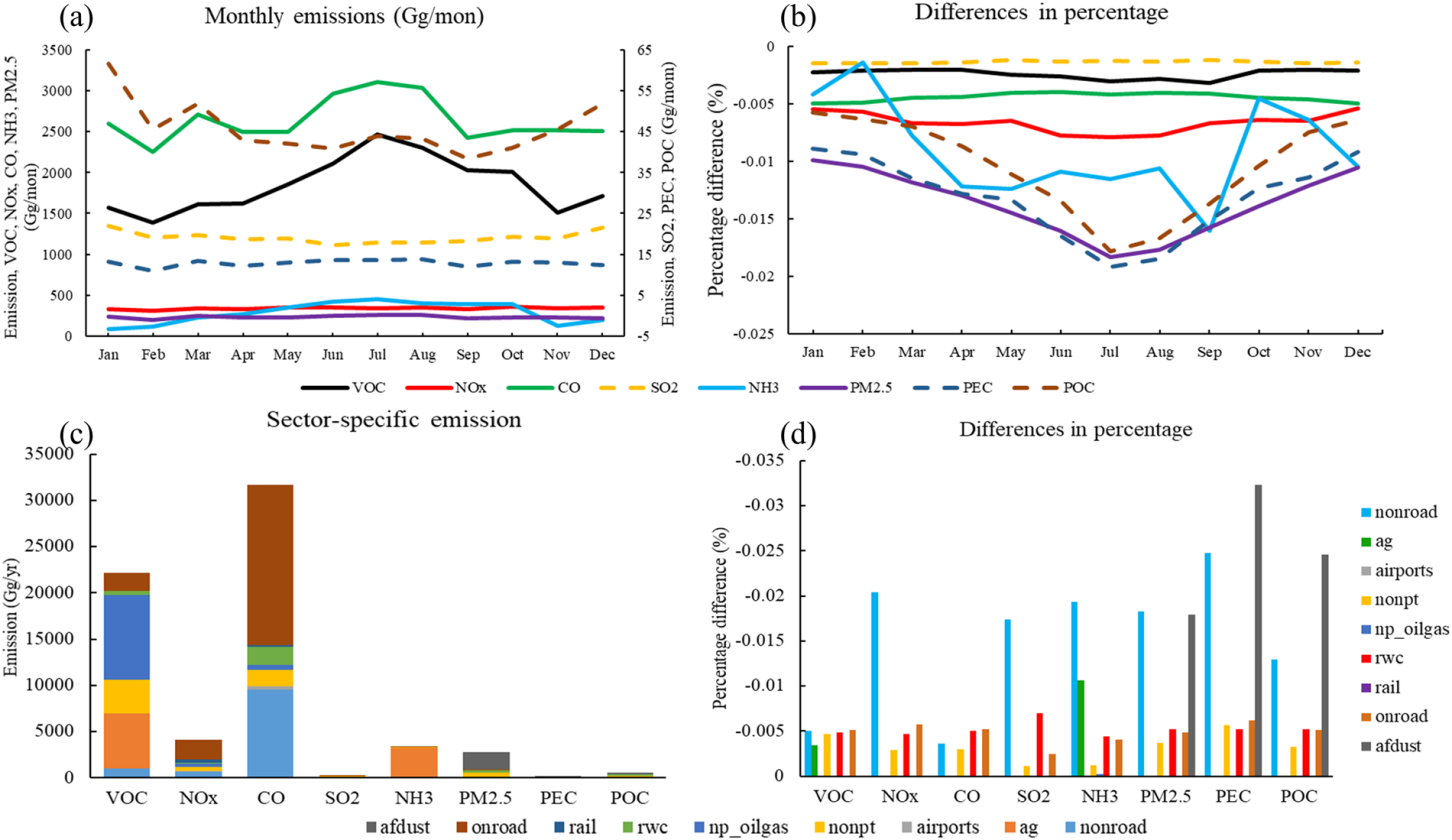
Temporal variations and sector contribution of seven representative species in NEMO and comparison to the NEI2017 benchmark dataset: (**a**) monthly variations of VOCs, NO_x_, CO, SO_2_, PM_2.5_, particulate organic carbon (POC) and particulate elemental carbon (PEC) emission in NEMO; (**b**) the difference (%) from the benchmark; (**c**) contribution by each emission sector (**c**) and (**d**) their differences (%) from the benchmark.

Here we compared the NEMO dataset against the 12 km × 12 km emission, generated using the spatial surrogates provided by US EPA in NEI2017, a benchmark dataset widely used in research and regulatory modeling. Figure 4 depicts the monthly emissions over CONUS of the NEMO dataset and the differences with those of 12 km. We found that the 1 km × 1 km emissions of each variable are almost identical to those of the benchmark dataset, although slightly lower (<−0.02%) than the latter. The differences between 1 km and 12 km datasets are more significant during summertime when the monthly emissions are higher than in other seasons (Fig. 4a). Figure 4 also shows the percentage differences of particulate matter are usually higher than those of the gases and the largest difference appears in black carbon (PEC) of July with a value of −0.02%. The sector-specific emissions in Fig. 4c,d show that most variables in the nonroad sector and particulate matters in the anthropogenic fugitive dust sector have larger underestimations. The difference in the emissions of other sectors are between 0.001% and 0.01%. In general, our dataset is consistent with the benchmark emissions.

### NO_x_ emissions over five large cities

**Fig. 5:**
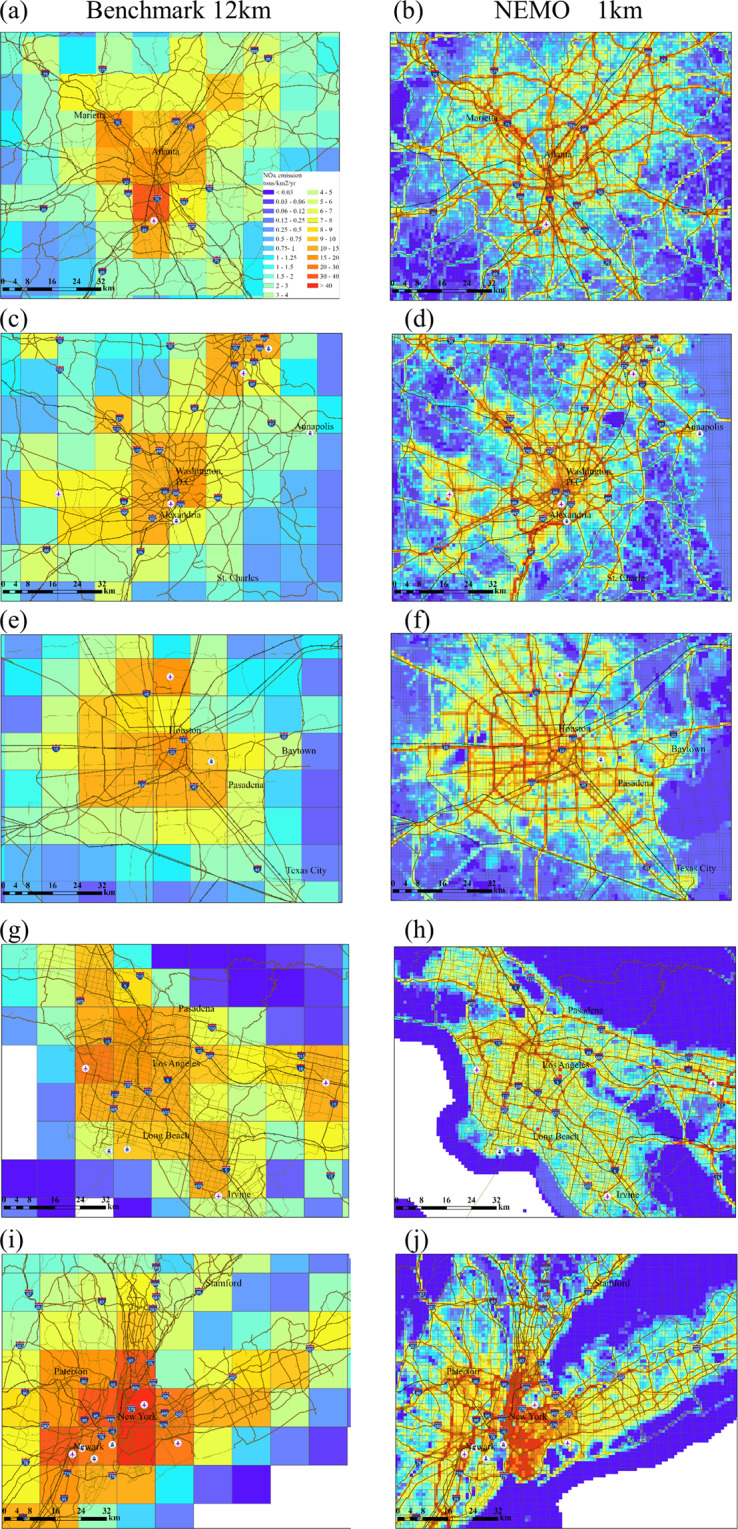
Surface anthropogenic NO_x_ emissions from the benchmark (12 km) and NEMO (1 km) datasets in five metropolitan cities, Atlanta (**a**,**b**), DC (**c**,**d**), Houston (**e**,**f**), Los Angeles (**g**,**h**) and New York City (**i**,**j**). The brown lines indicate the state/interstate roadways, the black lines with vertical short lines mean the railways, the symbols with plane indicate the locations of airports and the marks with ferry indicate the locations of ferries and ports.

Next, we compare the NO_x_ emissions over five metropolitan areas to that of the benchmark dataset. NO_x_ is a key precursor to tropospheric ozone and particulate nitrate. Figure 5 shows the annual emissions of NO_x_ from 12 km and 1 km dataset. We overlay the emission map with geographical information including roads, airports, ferries, and main cities as a measure to validate the accuracy of the spatial allocation. The results show that the NEMO dataset can capture high emissions in urban areas that follow the benchmark pattern. The 1 km distribution can also reflect the fine features of emissions over highways and other major roads. At airports, ultra-high NO_x_ emissions are shown at corresponding locations. In addition, the 1 km distributions create much clearer coast-pattern emissions over cities like New York City and Los Angeles compared to the benchmark. These results show that the spatial distribution of the 1 km emission dataset is more consistent with the geographical features in the real world. The increase of resolution (144 times finer than the benchmark) in comparison to the 12 km product provides the desirable information to map air pollutant emissions at neighborhood level.

## Usage Notes

The NEMO data are available in the NetCDF format at hourly, monthly and annual intervals. The shapefile format of NEMO is only available for the annual aggregated emissions, although finer temporal resolution can be generated from the NetCDF files. Each hourly emission file includes 5397 columns, 3177 rows, 35 gas species, 20 aerosol species, and 25 time steps which needs a longer time for processing. We recommend using double precision for data analysis and processing. For convenience, we also provide a web-based data portal^45^ to prepare anthropogenic emissions within the CONUS domain according to the user’s requirements.

## Supplementary information

SUPPLEMENTARY INFORMATION

Supplementary Information Table 1

## Data Availability

Code used for calculating monthly and annual emission is written in Fortran and available from Zenodo^46^. The Spatial Allocator version 4.4 and SMOKE version 4.7 are used for data processing which can be obtained from CMAS webpage^40^.
