# Supplementary material for: Neighborhood Emission Mapping Operation (NEMO): A 1-km anthropogenic emission dataset in the United States
Source: Sci Data. 2022 Nov 9;9:680. doi: 10.1038/s41597-022-01790-9 (PMC9646775; doi:10.1038/s41597-022-01790-9)
Supplement: Supplementary file 1 — SUPPLEMENTARY INFORMATION [file 41597_2022_1790_MOESM1_ESM.docx]

**Supplementary Material for**

**Neighborhood Emission Mapping Operation (NEMO): A 1-km anthropogenic emission dataset in the United States**

Siqi Ma^1^ and Daniel Q. Tong^1^

^1^ Department of Atmospheric, Oceanic and Earth Sciences, George Mason University, Fairfax, VA 22030, USA

Corresponding Authors: Daniel Q. Tong ([qtong@gmu.edu](mailto:qtong@gmu.edu)), Siqi Ma ([sma8@gmu.edu](mailto:sma8@gmu.edu))

Contents

[Supplementary Table 1. List of spatial surrogates, surrogate code and underlying geographical information used to generate these spatial surrogate ratios 2](#_Toc63324410)

**Supplementary Table 1. List of spatial surrogates, surrogate code and underlying geographical information used to generate these spatial surrogate ratios**

| **Surrogate name** | **Surrogate code** | **Geographical information maps used (shapefiles)** |
| --- | --- | --- |
| Population | 100 | US county boundary and American Community Survey (ACS) census-based population in 2014 |
| Housing | 110 | US county boundary and ACS housing in 2014 |
| Urban Housing | 131 | US county boundary and ACS housing in 2014 |
| Suburban Housing | 132 | US county boundary and ACS housing in 2014 |
| Rural Housing | 134 | US county boundary and ACS housing in 2014 |
| Housing Change | 137 | US county boundary and ACS housing change from 2010 to 2014 |
| Residential Heating - Natural Gas | 150 | US county boundary and ACS home heating fuels |
| Residential Heating - Wood | 160 | US county boundary and ACS home heating fuels |
| 0.5 Residential Heating - Wood plus 0.5 Low Intensity Residential | 165 | US county boundary |
| Residential Heating - Distillate Oil | 170 | US county boundary and ACS home heating fuels |
| Residential Heating - Coal | 180 | US county boundary and ACS home heating fuels |
| Residential Heating - LP Gas | 190 | US county boundary and ACS home heating fuels |
| Urban Restricted Road Miles | 201 | Highway Performance Monitoring System (HPMS) roadways in 2016 |
| Urban Restricted AADT | 202 | HPMS roadways 2016 |
| Extended Idle Locations | 205 | US county boundary and potential idling location |
| Rural Restricted Road Miles | 211 | HPMS roadways 2016 |
| Rural Restricted AADT | 212 | HPMS roadways 2016 |
| Urban Unrestricted Road Miles | 221 | HPMS roadways 2016 |
| Urban Unrestricted AADT | 222 | HPMS roadways 2016 |
| Rural Unrestricted Road Miles | 231 | HPMS roadways 2016 |
| Rural Unrestricted AADT | 232 | HPMS roadways 2016 |
| Total Road AADT | 239 | HPMS roadways 2016 |
| Total Road Miles | 240 | HPMS roadways 2016 |
| Total Restricted Road Miles | 241 | HPMS roadways 2016 |
| All Restricted AADT | 242 | HPMS roadways 2016 |
| Total Unrestricted Road Miles | 243 | HPMS roadways 2016 |
| All Unrestricted AADT | 244 | HPMS roadways 2016 |
| Intercity Bus Terminals | 258 | US county boundary and National Transportation Atlas Database (NTAD) bus terminals 2014 |
| Transit Bus Terminals | 259 | US county boundary and NTAD bus terminals 2014 |
| Total Railroad Miles | 260 | US county boundary and TIGER/Line rail length 2014 |
| NTAD Total Railroad Density | 261 | US county boundary and NTAD rail freight density 2014 |
| NTAD Class 1 2 3 Railroad Density | 271 | US county boundary and NTAD rail freight density 2014 |
| NTAD Amtrak Railroad Density | 272 | US county boundary and NTAD rail freight density 2014 |
| NTAD Commuter Railroad Density | 273 | US county boundary and NTAD rail freight density 2014 |
| ERTAC Rail Yards | 275 | US county boundary and Eastern Regional Technical Advisory Committee (ERTAC) rail yards |
| Class 2 and 3 Railroad Miles | 280 | US county boundary and TIGER/Line rail length 2014 |
| NLCD Low Intensity Development | 300 | US county boundary and National Land Cover Database (NLCD) land cover in 2011 |
| NLCD Med Intensity Development | 301 | US county boundary and NLCD land cover in 2011 |
| NLCD High Intensity Development | 302 | US county boundary and NLCD land cover in 2011 |
| NLCD Open Space | 303 | US county boundary and NLCD land cover in 2011 |
| NLCD Open + Low | 304 | US county boundary and NLCD land cover in 2011 |
| NLCD Low + Med | 305 | US county boundary and NLCD land cover in 2011 |
| NLCD Med + High | 306 | US county boundary and NLCD land cover in 2011 |
| NLCD All Development | 307 | US county boundary and NLCD land cover in 2011 |
| NLCD Low + Med + High | 308 | US county boundary and NLCD land cover in 2011 |
| NLCD Open + Low + Med | 309 | US county boundary and NLCD land cover in 2011 |
| NLCD Total Agriculture | 310 | US county boundary and NLCD land cover in 2011 |
| NLCD Pasture Land | 318 | US county boundary and NLCD land cover in 2011 |
| NLCD Crop Land | 319 | US county boundary and NLCD land cover in 2011 |
| NLCD Forest Land | 320 | US county boundary and NLCD land cover in 2011 |
| NLCD Recreational Land | 321 | US county boundary and NLCD land cover in 2011 |
| NLCD Land | 340 | US county boundary and NLCD land cover in 2011 |
| NLCD Water | 350 | National Transportation Atlas Database (NTAD) county and NLCD land cover in 2011 |
| Commercial Land | 500 | US county boundary and FEMA Building Footprints |
| Education | 506 | US county boundary and FEMA Building Footprints |
| Heavy Light Construction Industrial Land | 507 | US county boundary and FEMA Building Footprints |
| Commercial plus Industrial | 510 | US county boundary and FEMA Building Footprints |
| Commercial plus Institutional Land | 515 | US county boundary and FEMA Building Footprints |
| Commercial plus Industrial plus Institutional | 520 | US county boundary and FEMA Building Footprints |
| Golf Courses plus Institutional plus Industrial plus Commercial | 525 | US county boundary |
| Residential - Non-Institutional | 526 | US county boundary and FEMA Building Footprints |
| Single Family Residential | 527 | US county boundary and FEMA Building Footprints |
| Residential + Commercial + Industrial + Institutional + Government | 535 | US county boundary and FEMA Building Footprints |
| Retail Trade (COM1) | 540 | US county boundary and FEMA Building Footprints |
| Personal Repair (COM3) | 545 | US county boundary and FEMA Building Footprints |
| Professional/Technical (COM4) plus General Government (GOV1) | 555 | US county boundary and FEMA Building Footprints |
| Hospital (COM6) | 560 | US county boundary and FEMA Building Footprints |
| Light and High Tech Industrial (IND2 + IND5) | 575 | US county boundary and FEMA Building Footprints |
| Food Drug Chemical Industrial (IND3) | 580 | US county boundary and FEMA Building Footprints |
| Metals and Minerals Industrial (IND4) | 585 | US county boundary and FEMA Building Footprints |
| Heavy Industrial (IND1) | 590 | US county boundary and FEMA Building Footprints |
| Light Industrial (IND2) | 595 | US county boundary and FEMA Building Footprints |
| Industrial plus Institutional plus Hospitals | 596 | US county boundary and FEMA Building Footprints |
| Refineries and Tank Farms | 650 | US county boundary and Energy Information Administration (EIA) refineries and tank farms |
| Spud Count - CBM Wells | 670 | US county boundary and oil/gas production, well location and exploration statistics |
| Spud Count - Gas Wells | 671 | US county boundary and oil/gas production, well location and exploration statistics |
| Gas Production at Oil Wells | 672 | US county boundary and oil/gas production, well location and exploration statistics |
| Oil Production at CBM Wells | 673 | US county boundary and oil/gas production, well location and exploration statistics |
| Unconventional Well Completion Counts | 674 | US county boundary and oil/gas production, well location and exploration statistics |
| Well Count - All Producing | 676 | US county boundary and oil/gas production, well location and exploration statistics |
| Well Count - All Exploratory | 677 | US county boundary and oil/gas production, well location and exploration statistics |
| Completions at Gas Wells | 678 | US county boundary and oil/gas production, well location and exploration statistics |
| Completions at CBM Wells | 679 | US county boundary and oil/gas production, well location and exploration statistics |
| Spud Count - Oil Wells | 681 | US county boundary and oil/gas production, well location and exploration statistics |
| Produced Water at All Wells | 683 | US county boundary and oil/gas production, well location and exploration statistics |
| Completions at Oil Wells | 685 | US county boundary and oil/gas production, well location and exploration statistics |
| Completions at All Wells | 686 | US county boundary and oil/gas production, well location and exploration statistics |
| Feet Drilled at All Wells | 687 | US county boundary and oil/gas production, well location and exploration statistics |
| Well Counts - CBM Wells | 691 | US county boundary and oil/gas production, well location and exploration statistics |
| Spud Count - All Wells | 692 | US county boundary and oil/gas production, well location and exploration statistics |
| Well Count - All Wells | 693 | US county boundary and oil/gas production, well location and exploration statistics |
| Oil Production at Oil Wells | 694 | US county boundary and oil/gas production, well location and exploration statistics |
| Well Count - Oil Wells | 695 | US county boundary and oil/gas production, well location and exploration statistics |
| Gas Production at Gas Wells | 696 | US county boundary and oil/gas production, well location and exploration statistics |
| Oil Production at Gas Wells | 697 | US county boundary and oil/gas production, well location and exploration statistics |
| Well Count - Gas Wells | 698 | US county boundary and oil/gas production, well location and exploration statistics |
| Gas Production at CBM Wells | 699 | US county boundary and oil/gas production, well location and exploration statistics |
| Airport Points | 710 | US county boundary and airport point |
| Airport Areas | 711 | US county boundary and airport area |
| Port Areas | 801 | US county boundary and marine port areas |
| Offshore Shipping Area | 805 | Offshore commercial shipping |
| Offshore Shipping NEI2014 Activity | 806 | Offshore commercial shipping |
| Navigable Waterway Miles | 807 | NTAD Waterways and marine port areas |
| 2013 Shipping Density | 808 | Offshore commercial shipping and vessel density |
| Ports NEI2014 Activity | 820 | NTAD county and marine port areas |
| Golf Courses | 850 | US county boundary and point of Interest (POI) Factory golf courses |
| Mines | 860 | US county boundary and USGS Mineral Resources Data System (MRDS) point locations |
| Commercial Timber | 890 | US county boundary and U.S. Forest Service commercial timber |
